# Gastric Cancer With Situs Inversus Totalis: A Case Report With More Than Five Years of Follow-Up

**DOI:** 10.7759/cureus.39512

**Published:** 2023-05-26

**Authors:** Hussain A Al Jabran, Mamdouh Alnahawi, Mousa J Alhaddad

**Affiliations:** 1 Department of Surgery, Almoosa Specialist Hospital, Alahsa, SAU; 2 Department of Internal Medicine, Dammam Medical Complex, Dammam, SAU

**Keywords:** saudi arabia, gastrectomy, stomach cancer, dextrocardia, situs inversus

## Abstract

In Saudi Arabia, gastric cancer is a major health burden, ranking as the thirteenth most common cancer type. Situs inversus totalis (SIT), which is a very rare congenital anomaly, refers to a completely reversed location of the abdominal and thoracic organs (mirror image of the normal). Here, we present the first reported case of gastric cancer in an SIT patient in Saudi Arabia and the Gulf Cooperation Council (GCC) countries, and highlight the challenges faced by the surgical team in the removal of such cancer in this patient population.

## Introduction

Gastric cancer remains one of the most important cancers worldwide, as it ranks as the fifth most common cancer and the fourth leading cause of cancer death globally in 2020 [1].

In Saudi Arabia, gastric cancer is a major health burden, ranking as the thirteenth most common cancer type in the country [2] with a total of 4,066 registered gastric cancer cases in the Saudi Cancer Registry between 2004 and 2017 [3]. The incidence of gastric cancer in Saudi Arabia is steadily dropping year by year owing to the increase in hygiene standards and Helicobacter pylori eradication [3], with an age-standardized incidence rate of 2.7 cases per 100,000 individuals in 2010 [3] compared to 2.1 cases per 100,000 individuals in 2020 [2].

Situs inversus totalis (SIT) refers to a completely reversed location of the abdominal and thoracic organs [4]. The heart is located on the right side of the chest (dextrocardia) with a complete reversal of the heart chambers. The normal pulmonary anatomy is also reversed, so the left lung has three lobes and the right lung has two lobes. In addition, the liver and gallbladder are located on the left side of the abdomen, whereas the spleen and stomach are located on the right side. The remaining internal structures are also a mirror image of the normal. This congenital anomaly is very rare with an incidence of one case in 10,000 births [4].

Here, we present a case of gastric cancer and highlight the challenges faced by the surgical team in the removal of such cancer in SIT patients. In the literature, seven case reports could be found that described operations in adult SIT patients in Saudi Arabia with five reports describing cholecystectomies [5-9] and two reports describing bariatric surgeries [10,11], making this case report the first describing gastrectomy - and the first oncological procedure - in SIT patients in Saudi Arabia and the Gulf Cooperation Council (GCC) countries.

## Case presentation

A 33-year-old male patient who was known to have SIT presented in December 2017 to a gastrointestinal clinic complaining of recurrent abdominal pains for the preceding three months. His pains were associated with nausea and abdominal bloating.

The patient was diagnosed to have SIT when he was evaluated in another center by abdominal ultrasound and chest radiograph for abdominal pain during his childhood. He had no other known comorbid conditions. He was not a smoker and had no history of alcohol intake. He was working in a gas fuel station. There was no history of gastric malignancy in his family.

His weight and height were 71 kg and 164 cm, respectively, with a body mass index (BMI) of 26.4 kg/m^2^. His examination was unremarkable apart from a minimal epigastric tenderness. His hemoglobin was 14.6 g/dL. The white blood cell (WBC) count was 4.6 ×10^9^/L. The platelet count was 307 ×10^9^/L. His renal and liver function tests were normal with a negative stool occult blood test.

**Figure 1:**
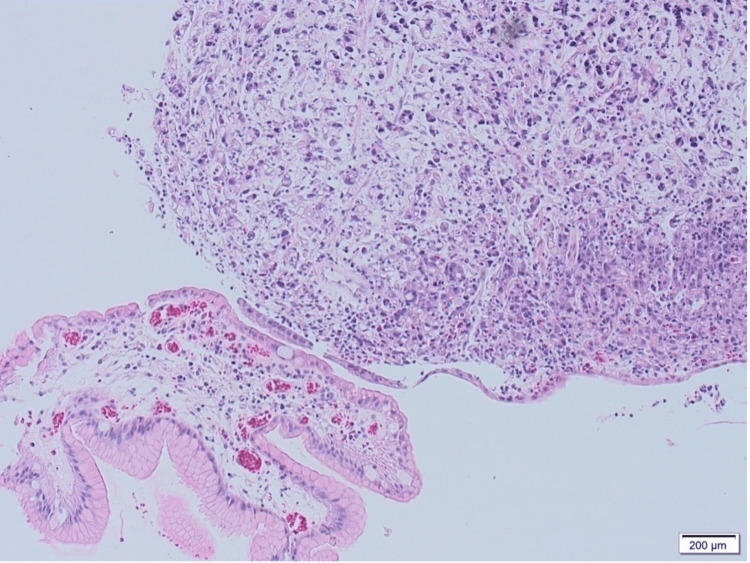
A histopathology slide showing a poorly differentiated signet ring cell gastric adenocarcinoma (hematoxylin and eosin stain with 10x magnification)

 The patient tested positive for Helicobacter pylori (H. pylori) urea breath test. An esophagogastroduodenoscopy (EGD) showed a large clean-based ulcer at the incisura angularis of the stomach with no other abnormal pathological findings. The ulcer was biopsied, and the histopathology report revealed a poorly differentiated adenocarcinoma with a signet ring component (Figure 1).

**Figure 2:**
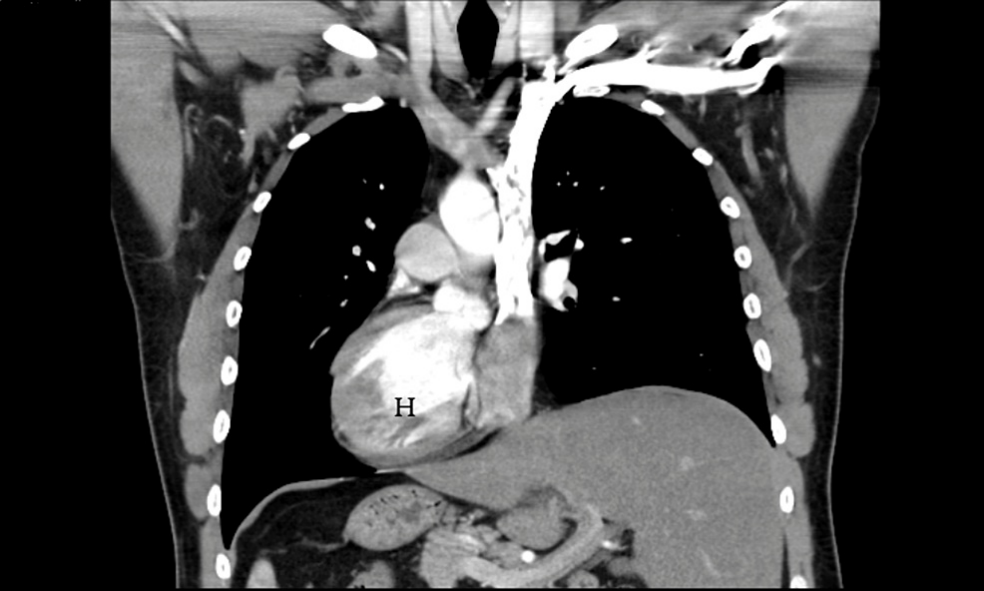
A CT scan of the chest showing the heart (H) lying in the right side (dextrocardia)

**Figure 3:**
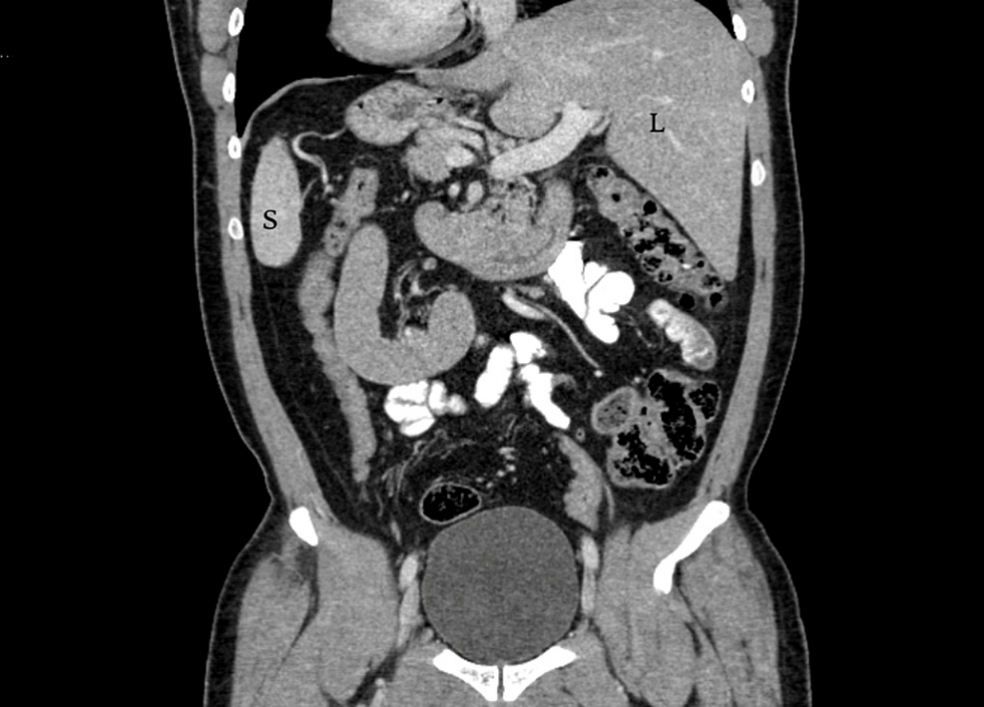
A Coronal view of an abdominopelvic CT scan showing a reversed location of the abdominal organs with the liver (L) positioned in the left side and the spleen (S) being in the right side

**Figure 4:**
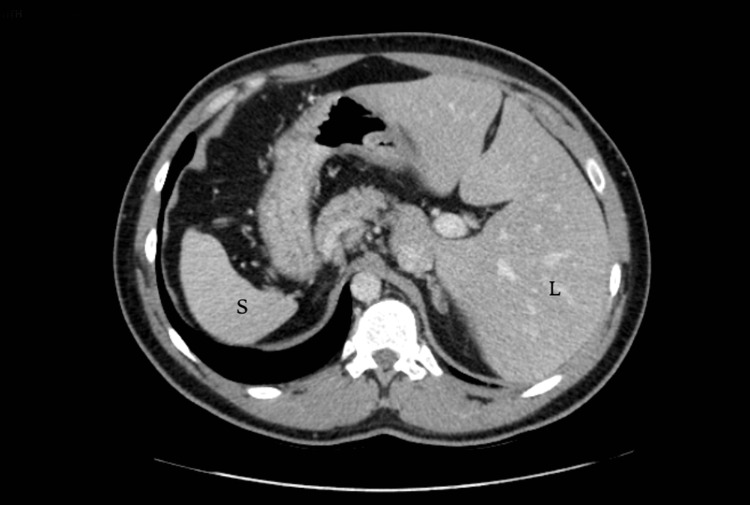
An axial view of an abdominopelvic CT scan showing a reversed location of the abdominal organs with the liver (L) positioned in the left side and the spleen (S) being in the right side

The serum tumor markers carcinoembryonic antigen (CEA) and carbohydrate antigen 19-9 (CA 19-9) were not elevated. A chest, abdomen, and pelvis computed tomography (CT) scan showed a complete transposition of the heart and the abdominal viscera, confirming SIT (Figures 2-4).

The primary gastric tumor was not seen in the CT images, but a prominent gastro-esophageal junction lymph node was found, measuring 1.7 cm * 0.9 cm. Otherwise, no lymphadenopathy or distant metastases were noted in the abdomen and pelvis. A fluorodeoxyglucose (FDG)-positron emission tomography (PET) scan revealed a hypermetabolic lesion in the distal posterior gastric wall with a maximum standard uptake value (SUVmax) of 7.16 g/mL with a positive non-specific low FDG avid uptake in the gastro-esophageal lymph nodes to the left of cardia that was interpreted as inflammatory reactive lymph nodes rather than lymph node metastasis. No liver, bone, or peritoneal metastasis were appreciated. Endoscopic ultrasound was not performed, as it was neither available in our center at that time nor easily accessible in nearby hospitals.

The case was discussed in the tumor board meeting, and the decision was to go for upfront surgery. An open laparotomy was performed due to the expected difficulties of the unusual mirror image anatomy in SIT. An esophagogastroduodenoscopy (EGD) was also done intraoperatively by a gastroenterology consultant, which helped confirm and localize the gastric lesions. The stomach and spleen were located on the right side of the abdomen, and the gallbladder, liver, cecum, and appendix were located on the left side. A subtotal gastrectomy with Roux-en-Y gastrojejunostomy was performed with a D2 lymph node dissection. No vascular anomalies were observed, and no intraoperative complications were encountered.

**Figure 5:**
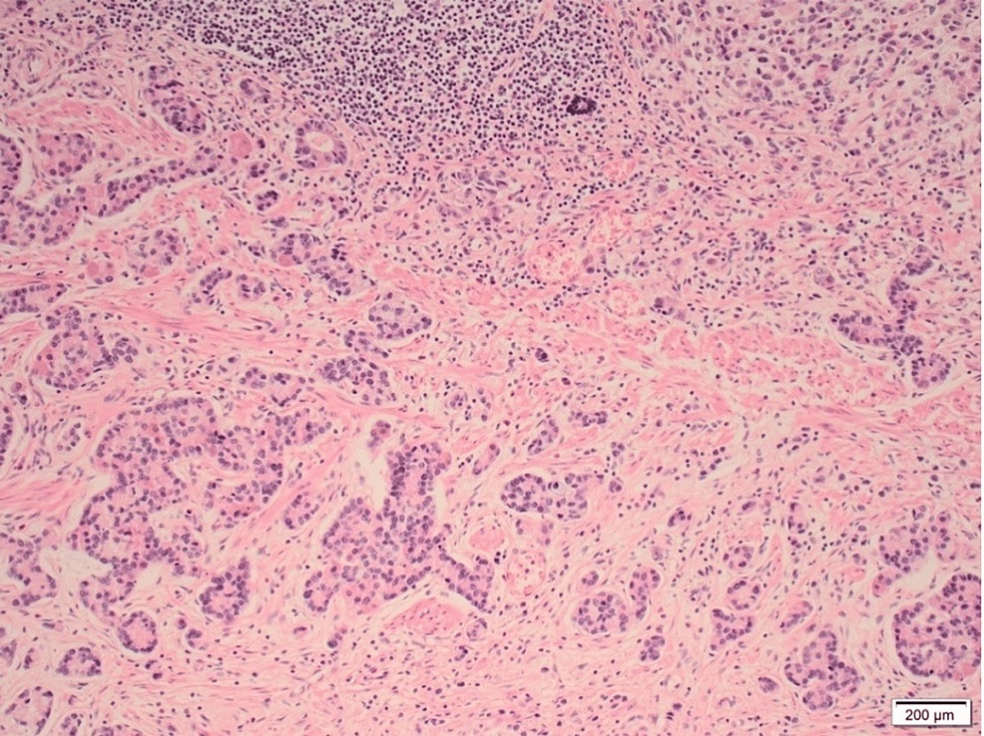
A pathological exam of the resected lesion showing poorly differentiated adenocarcinoma with a signet ring component (hematoxylin and eosin stain with 4x magnification)

The final histopathology from the resected stomach was consistent with gastric cancer of a poorly differentiated adenocarcinoma with a signet ring cell component (Figure 5). The tumor invaded the submucosa with 12 involved lymph nodes (out of a total of 24 removed nodes) with a pathological stage of pT1bN3aM0 corresponding to a stage group of IIB.

Following the operation, the patient stayed in the hospital mainly for postoperative pain management. He was discharged on Day 5 postoperative with no postoperative complications. He was then followed by the medical oncology team for adjuvant chemotherapy. He uneventfully completed nine cycles of the capecitabine plus oxaliplatin (XELOX) protocol. He was followed for more than five years in the outpatient department by surveillance CT scans of the chest, abdomen, and pelvis every six months to one year without evidence of cancer recurrence.

## Discussion

Upon searching PubMed for "(situs inversus) AND (cancer)" on April 14, 2023, and reviewing the results, 44 case reports were found about gastric cancers in SIT patients. The vast majority of the cases were reported in China, Japan, and Korea. This is mostly because Eastern Asia has the highest age-standardized incidence rate of gastric malignancies at 32.5 cases per 100,000 individuals [12]. As a comparison, the rates are 11.4 and 5.4 cases per 100,000 individuals in Western Asia and Northern Africa, respectively [12]. The closest countries to Saudi Arabia where cases of gastric cancers were reported in SIT patients are Morocco [13] and Turkey [14].

For SIT patients with gastric cancer, the treatment modality is no different than that for non-SIT gastric cancer patients. However, SIT can pose a challenge during surgery because of the extraordinary anatomy of these patients, especially with dissecting the peri-gastric vessels. Vascular anomalies were seen in 47.6% of SIT patients with gastric cancer [14]. Examples of these anomalies include the left hepatic artery originating from the superior mesenteric artery [15], the left gastric artery branching directly from the aorta [16], and the left gastric artery having double branches [17]. Thus, surgeries in this population need to be carefully planned preoperatively, and CT angiograms might be additive in these settings [14-17].

The reversed anatomy in SIT patients, along with the associated vascular anomalies, was linked to an increased operative time [10,18]. There is also a non-consensus on the proper positioning of the operator and assistant beside the patient in the operative room, especially during laparoscopic surgeries, with some surgeons recommending reversing the operator and assistant positions to go along with the reversed anatomy [14]. In our case, it has been a conflicting issue whether to stand to the right of the patient or to his left side, but the surgeon and assistant worked in their standard places (with the surgeon positioning on the right side of the patient and the assistant standing on his left side) and completed the surgical procedure successfully.

The patient being young and healthy with no previous abdominal surgeries, the careful preoperative planning, the experience of the operating hepatobiliary and surgical oncology surgeon, the open surgical approach, the intraoperative EGD, the presence of a large screen in the operating room to allow for reviewing the CT images during the procedure and the absence of vascular abnormalities all contributed to the performance of the surgery with no complications.

In SIT patients, both open and minimally invasive gastrectomy procedures have been performed successfully with a very low rate of postoperative complications, especially if they were performed in high-volume centers by very experienced surgeons [14,18]. If a laparoscopic approach is chosen, the different ports should be positioned at mirror image sites of those in the usual patients [7]. The dissection process was reported to be difficult for right-handed surgeons [7], and a decision to convert to open surgery should be carried out if the surgeon is not fully at ease with completing it laparoscopically. Of note, the operative time and the hospital stay were comparable between laparoscopic and robotic procedures, but robotic gastrectomy procedures caused less bleeding. However, the magnitude of the difference is small (around 32.5 ml) [18].

## Conclusions

SIT is a rare congenital anomaly that poses unique challenges during surgical management. Preoperative planning, including imaging and careful consideration of surgical techniques, is essential for successful outcomes in SIT patients with gastric cancer. The use of laparoscopic and robotic techniques has been shown to be safe and effective in this patient population. Further research is needed to improve the understanding of the etiology and optimal management of SIT patients with gastric cancer.
